# TQFL19, a Novel Derivative of Thymoquinone (TQ), Plays an Essential Role by Inhibiting Cell Growth, Metastasis, and Invasion in Triple-Negative Breast Cancer

**DOI:** 10.3390/molecules30040773

**Published:** 2025-02-07

**Authors:** Ali El-Far, Xiaoyan Liu, Ting Xiao, Jun Du, Xinwei Du, Chunli Wei, Jingliang Cheng, Hui Zou, Junjiang Fu

**Affiliations:** 1 Key Laboratory of Epigenetics and Oncology, The Research Center for Preclinical Medicine, Southwest Medical University, Luzhou 646000, China; alih.elfar2024@swmu.edu.cn (A.E.-F.); jczxlxy@swmu.edu.cn (X.L.); xiaoting@swmu.edu.cn (T.X.); weichunli2015@swmu.edu.cn (C.W.); jingliangc@swmu.edu.cn (J.C.); 2 Department of Chemistry, Southwest Medical University, Luzhou 646000, China; dujun@swmu.edu.cn; 3 School of Pharmacy, Southwest Medical University, Luzhou 646000, China; 18381766313@163.com; 4 Key Laboratory of Study and Discovery of Small Targeted Molecules of Hunan Province, School of Medicine, Hunan Normal University, Changsha 410013, China

**Keywords:** thymoquinone, TQ derivative, TQFL19, triple-negative breast cancer, metastasis, anticancer

## Abstract

Breast cancer (BC) is a significant public health concern globally. Triple-negative breast cancer (TNBC) is considered the most challenging type, as it is defined by an absence of estrogen and progesterone receptor expression, along with a lack of HER2 overexpression. In the current study, we developed a novel thymoquinone (TQ), TQFL19, to control TNBC progression. **Purpose:** The current study aimed to investigate the anticancer potential of a newly synthesized TQFL19 against TNBC. **Study design:** To achieve our research goals, we meticulously developed both in vitro and in vivo studies focused on TNBC cell growth, metastasis, and invasion. **Results:** Characterization and ADMET properties prediction of TQFL19 were first performed before treating TNBC cells. TQFL19 exhibited more potent cytotoxicity than TQ against 4T1, BT-549, and MDA-MB-231 cells and induced apoptosis of 4T1 and MDA-MB-231, besides cell cycle arrest of MDA-MB-231. In vivo mice allograft of 4T1 revealed the ability of TQFL19 to hinder the growth, migration, and metastasis of TNBC cells. **Conclusions:** The results suggest that TQFL19 potentially inhibited TNBC growth, metastasis, and invasion. The results conclude that TQFL19 could be a viable candidate for TNBC therapy.

## 1. Introduction

Breast cancer (BC) is the primary cause of cancer-related deaths in women globally, with approximately 2.3 million new diagnoses and 685,000 fatalities reported in 2020 [1]. BC cells show different metabolic phenotypes influenced by internal factors like *MYC* amplification and mutations in *PIK3CA* and *TP53*, as well as external factors such as hypoxia, oxidative stress, and acidosis. These elements lead to varying metabolic reprogramming in metastatic BC [2]. BC is a diverse disease with four main molecular subtypes: luminal A, luminal B, HER2-positive, and triple-negative breast cancer (TNBC). Each subtype has distinct levels of proliferation and metastasis, as well as unique metabolic characteristics [3]. TNBC is the sole subtype of BC without targeted therapies, accounting for 15% to 20% of all cases that are characterized by the lack of hormonal receptor expression, and HER2 overexpression carries the worst prognosis [4]. Also, these properties lead to the ineffectiveness of the main targeted treatments for TNBC patients [5]. In spite of various efforts to find targetable therapies, chemotherapy continues to be the primary systemic treatment for TNBC [6].

BC treatment options are determined by grade, stage, and molecular subtypes to provide the most personalized, safe, and effective care [7]. For non-metastatic BC, the treatment approach includes surgically removing the tumor, followed by radiotherapy—either before (neoadjuvant) or after (adjuvant) the surgery—and systemic treatments such as chemotherapy and targeted therapies [7,8,9]. Targeted treatments for BC involve endocrine therapy for hormone receptor-positive (HR+) cases and antiHER2 therapies for HER2-positive cases. However, TNBC has no targeted options available. In metastatic BC, the main goal is to manage tumor spread, as it remains incurable, using comparable systemic therapies [8]. New strategies for BC treatment include immunotherapy, conjugated antibodies, and natural products [10].

Natural products have been extensively used for cancer therapy, especially BC [11]. For example, camptothecin and taxol are the two most successful examples of natural products with anticancer potential [12,13]. Thymoquinone (TQ), a bioactive compound derived from the oil of *Nigella sativa* seeds, has been widely researched for its potential anticancer properties across multiple cancer types [14]. TQ-induced apoptosis and reduced BC cell proliferation, migration, and invasion [15,16,17]. Because of this anticancer potential of TQ, several studies tried to boost this power by producing TQ nanoformulation to enhance the bioavailability and targeting of cancer cells [18].

In our earlier research, we created a new derivative of TQ, known as TQFL12, which was more effective than TQ in inhibiting the metastasis and invasion of TNBC by enhancing the activation of the AMPK/ACC pathway [19]. The current study aimed to synthesize another new TQ derivative, TQFL19, and investigate its anticancer effect on TNBC. TQFL19 exhibited more potent cytotoxicity than TQ against 4T1, BT-549, and MDA-MB-231 cells and induced apoptosis of 4T1 and MDA-MB-231, besides cell cycle arrest of MDA-MB-231. In vivo mice allograft of 4T1 revealed the ability of TQFL19 to hinder the growth, migration, and metastasis of TNBC cells. TQFL19 could be a viable candidate for anticancer treatment for TNBC.

## 2. Results

### 2.1. Synthesis of TQFL19

**Figure 1:**
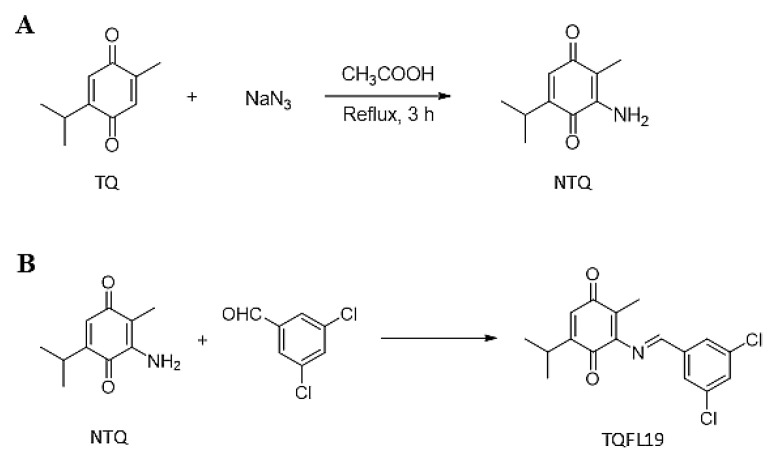
Synthesis and structure for the novel compound TQFL19. (**A**) Synthetic route of NTQ: TQ reacts with NaN3 to form NTQ. NTQ: 3-amino-5-isopropyl-2-methylcyclohexa-2,5-diene-1,4-dione. (**B**) Synthetic route of TQFL19: NTQ reacts with 3, 5-dichlorobenzaldehyde to form TQFL19. TQFL19, (E)-3-((3,5-dichlorobenzylidene) amino)-5-isopropyl-2-methylcyclohexa-2,5-diene-1,4-dione. The structure of TQFL19 is presented in panel B.

Synthesis of TQFL19 started from TQ to obtain 3-amino-thymoquinone (NTQ) with a yield of 65% (1.16 g) (Figure 1A). The NTQ is a red solid. Then, NTQ was stirred continuously in absolute ethanol and concentrated hydrochloric acid as a catalyst to obtain TQFL19 (Figure 1B). TQFL19 is a light-yellow solid with the molecular formula C_17_H_15_Cl_2_NO_2_. Results in Figure 1B also revealed the chemical structure of TQFL19, named (E)-3-((3,5-dichlorobenzylidene) amino)-5-isopropyl-2-methylcyclohexa-2,5-diene-1,4-dione.

### 2.2. Characterization and Purity Analyses

By NMR, the characterization of compound NTQ is as follows: ^1^H NMR (500 MHz, DMSO-*d*_6_): 1.05 (6H, d), 1.72 (3H, s), 2.86 (1H, m), 6.28 (1H, s), 6.48 (2H, s). ^13^C NMR (125 MHz, DMSO-*d*_6_): 9.1, 21.5, 26.3, 106.7, 132.4, 145.6, 149.4, 184.0, 185.2.

**Figure 2:**
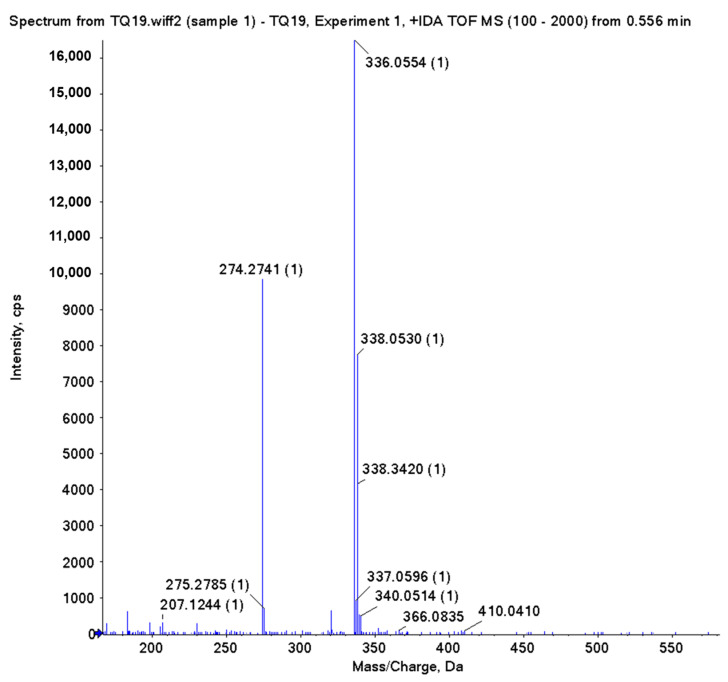
HRESIMS spectrum of the novel compound TQFL19. TQ-19 means TQFL19. Each experiment was repeated three times.

The NMR and HRESIMS data of TQFL19 were listed as follows. ^1^H NMR (400 MHz, DMSO-*d*_6_): *δ* 9.25 (s, 1H), 8.01 (d, *J* = 1.8 Hz, 2H), 7.80 (t, *J* = 1.8 Hz, 1H), 6.78 (s, 1H), 3.31–3.21 (m, 1H), 2.30 (s, 3H), 1.32 (d, *J* = 6.8 Hz, 6H) (Figure S1). ^13^C NMR (100 MHz, DMSO-*d*_6_): *δ* 158.8, 152.5, 141.9, 141.4, 135.0, 130.5, 130.1, 128.4, 125.1, 111.9, 111.4, 28.6, 22.5, 10.0 (Figure S2). HRESIMS (*m*/*z*, ESI) calcd for C_17_H_16_Cl_2_NO_2_ [M+H]^+^: 336.0553; found 336.0554 (Figure 2).

### 2.3. Prediction Results of ADMET Properties

The findings in Table 1 indicate that TQ and TQFL19 share similar ADMET properties, although TQFL19 exhibits lower solubility compared to TQ. Specifically, TQ has a water solubility of −3.021 (log mol/L) and a blood–brain barrier permeability of 0.006 (log BB). At the same time, TQFL19 shows a water solubility of −5.902 (log mol/L) and a blood–brain barrier permeability of 0.639 (log BB). Both TQ and TQFL19 were found to be non-mutagenic, according to the Ames test. Moreover, TQFL19 exhibited additional H-acceptor, two rotatable bonds, and one aromatic ring in its molecular structure than TQ. Regarding pharmacokinetics, TQ and TQFL19 exhibited high gastrointestinal absorption and different effects on cytochrome P450 enzymes.

### 2.4. Cytotoxicity and IC50s of TQ and TQFL19

The IC50 values of TQFL19 against 4T1 cancer cells were measured to be 26.169 μM after 24 h of exposure and decreased to 15.478 μM after 48 h. In contrast, the IC50 values for TQ were significantly higher, recorded at 72.05 μM at 24 h and 32.916 μM at 48 h. This suggests that TQFL19 exhibits a stronger potency against 4T1 cells over time than TQ (Table 2). TQFL19 is more effective than TQ against certain cancer cells. For BT-549 cells, TQFL19 has an IC50 of 44.271 μM after 24 h and 37.222 μM after 48 h. In comparison, TQ has an IC50 greater than 80 μM at both 24 and 48 h.

For MDA-MB-231 cells, TQFL19 shows an IC50 of 28.15 μM at 24 h and 27.30 μM at 48 h, while TQ has IC50 values of 48.76 μM at 24 h and 73.21 μM at 48 h.

TQFL19 also works against MDA-MB-468 cells, with IC50 values of 57.23 μM at 24 h and 40 μM at 48 h. In contrast, TQ has IC50 values greater than 100 μM at 24 h and 83.16 μM at 48 h.

**Figure 3:**
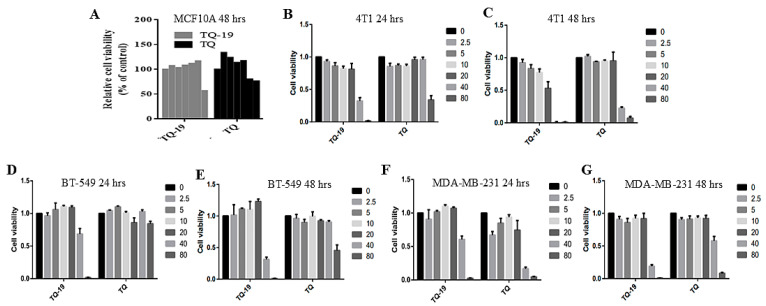
Relative cell viability of cells treated with TQFL19. (**A**) Relative cell viability of MCF10A cells treated with TQFL19 for 48 h. (**B**) Relative cell viability of 4T1 cells treated with TQFL19 for 24 h. (**C**) Relative cell viability of 4T1 cells treated with TQFL19 for 48 h. (**D**) Relative cell viability of BT-549 cells treated with TQFL19 for 24 h. (**E**) Relative cell viability of BT-549 cells treated with TQFL19 for 48 h. (**F**) Relative cell viability of MDA-MB-231 cells treated with TQFL19 for 24 h. (**G**) Relative cell viability of MDA-MB-231 cells treated with TQFL19 for 48 h. TQ-19 means TQFL19. TQ-19 means TQFL19. Each experiment was repeated three times.

Furthermore, TQFL19 exhibited no cytotoxicity for MCF10A 48 h until 40 μM, while TQ until 20 μM (Figure 3A). Compared with TQ, TQFL19 induced cytotoxicity in 4T1 (Figure 3B,C) and BT-549 (Figure 3D,E) cells at lower concentrations at 24 and 48 h. Meanwhile, for MDA-MB-231, TQFL19 induced more cytotoxicity than TQ at 48 h, not 24 h (Figure 3F,G).

Our previous results indicated that TQFL12 exhibited an IC50 of 20.241 μM against 4T1 at 24 h, while it had IC50s of 27.686 and 31.613 μM against BT549 and MDA-MB-231, respectively [19]. These data indicate that 4T1 and BT549 cells exhibited more sensitivity to TQFL12 than TQFL19, while MDA-MB-231 is more sensitive to TQFL19. We must study their actions closely to understand how TQFL12 and TQFL19 work in these cells.

### 2.5. Apoptosis and Cell Cycle Effects of TQFL19

**Figure 4:**
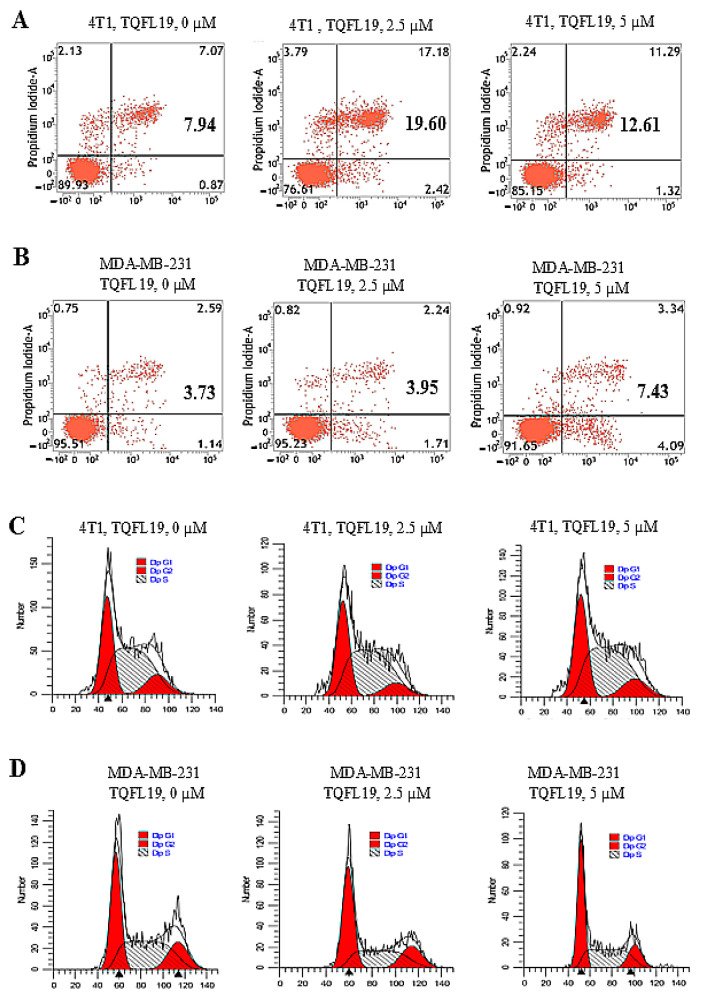
Apoptosis and cell cycle analyses. (**A**) Annexin-V positive 4T1 cells treated with TQFL19 at concentrations of 0, 2.5, and 5 µM. (**B**) Annexin-V positive MDA-MB-231 cells treated with TQFL19 at concentrations of 0, 2.5, and 5 µM. (**C**) Cell cycle analysis of 4T1 cells treated with TQFL19 at concentrations of 0, 2.5, and 5 µM. (**D**) Cell cycle analysis of MDA-MB-231 cells treated with TQFL19 at concentrations of 0, 2.5, and 5 µM. Values in A and B panels (7.94, 19.60, 12.61, 3.73, 3.95, and 7.43) are the sum of early apoptosis (lower right) and late apoptosis (upper right).

Apoptosis is determined by the percentages of cells that exhibited positive annexin-V, as shown in (Figure 4A,B). Both 4T1 (Figure 4A) and MDA-MB-231 (Figure 4B) exhibited apoptosis when treated with TQFL19 by concentrations of 2.5 and 5 μM.

Cell cycle analysis data illustrated in Figure 4C exhibited no changes in cell cycle phases in 4T1 treated with TQFL19. On the other hand, TQFL19 induced a 10% change in the cell cycle-G1-S phase of MDA-MB-231 at a concentration of 5 μM (Figure 4D).

### 2.6. TQFL19 Inhibits Cell Growth, Migration, and Invasion In Vitro

**Figure 5:**
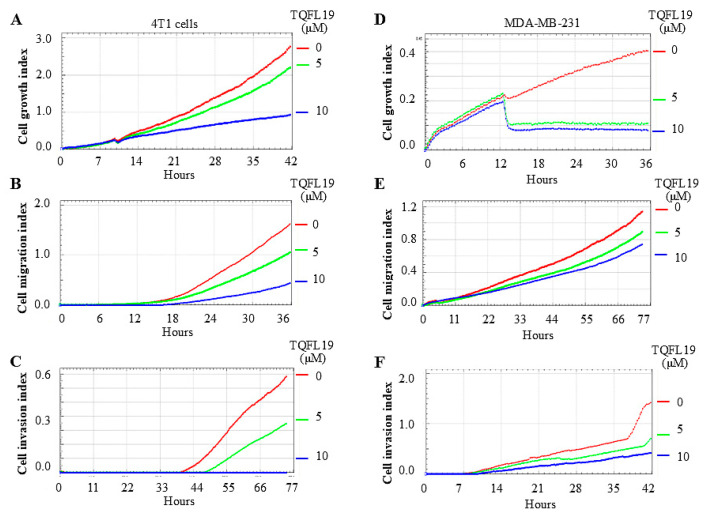
Breast cell growth, migration, and invasion indices. (**A**) Cell growth index of 4T1 cells treated with TQFL19 at concentrations of 0, 5, and 10 µM. (**B**) Cell migration index of 4T1 cells treated with TQFL19 at concentrations of 0, 5, and 10 µM. (**C**) Cell invasion index of 4T1 cells treated with TQFL19 at concentrations of 0, 5, and 10 µM. (**D**) Cell growth index of MDA-MB-231 cells treated with TQFL19 at concentrations of 0, 5, and 10 µM. (**E**) Cell migration index of MDA-MB-231 cells treated with TQFL19 at concentrations of 0, 5, and 10 µM. (**F**) Cell invasion index of MDA-MB-231 cells treated with TQFL19 at concentrations of 0, 5, and 10 µM. Each experiment was repeated three times.

**Figure 6:**
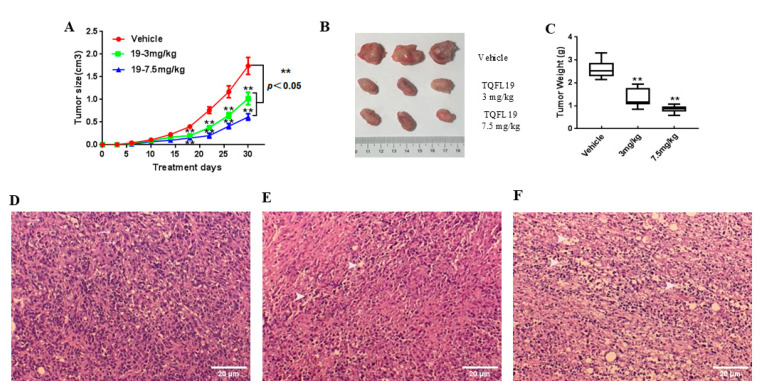
Mouse allograft. (**A**) Tumor size (cm^3^) of allograft 4T1 cells in control and mice treated with TQFL19 at concentrations of 3 and 7.5 mg/kg body weight. (**B**) Tumor weight (g) of allograft 4T1 cells in control and mice treated with TQFL19 at concentrations of 3 and 7.5 mg/kg body weight. (**C**) Quantitative results from panel B. (**D**) Photomicrograph of tumor mass of control group stained with H&E. Carcinoma with multiple mitosis featuring high mitotic figures (arrows). 400×. The bottom panel indicates the enlarged image from the top panel. (**E**) Photomicrograph of tumor mass of TQFL19-treated group (3 mg/kg body weight) stained with H&E. Carcinoma with few mitosis with multifocal necrosis (arrowheads). 400×. The bottom panel indicates the enlarged image from the top panel. (**F**) Photomicrograph of tumor mass of TQFL19-treated group (7.5 mg/kg body weight) stained with H&E: Carcinoma without mitosis with diffuse necrosis (arrowheads). 400×. ** *p* < 0.01. Scale bar = 20 μm.

In our research, we performed experiments to examine how TQFL19 treatment affects the growth, migration, and invasive characteristics of 4T1 and MDA-MB-231 cells. Our findings indicated a significant reduction in cell growth, migration, and invasion in both 4T1 (Figure 5A–C) and MDA-MB-231 (Figure 5D–F) cells following treatment with TQFL19 at concentrations of 5 and 10 μM. Furthermore, this decrease was found to be dependent on the dosage applied. The sudden downfall of cell growth values in Figure 6A indicates that the 4T1 cells exhibited a rapid response to TQFL19.

### 2.7. Mouse Allograft Assessment

Data represented in Figure 6A,B showed a significant decrease (*p* < 0.01) in tumor sizes in mice groups treated with TQFL19 at concentrations of 3 and 7.5 mg/kg compared with control. Also, mice treated with 3 and 7.5 mg/kg of TQFL19 exhibited significant reduction (*p* < 0.01) in tumor weight compared with control (Figure 6C).

Histopathological results in Figure 6D showed the photomicrograph of the tumor mass of the control group stained with H&E. It represents a carcinoma with multiple mitoses featuring high mitotic figures (Figure 6D, arrows). Figure 6E illustrates the photomicrograph of the tumor mass of the TQFL19-treated group (3 mg/kg body weight) stained with H&E (Figure 6E, arrowheads). The carcinoma exhibited few mitosis with multifocal necrosis. The photomicrograph of tumor mass of the TQFL19-treated group with 7.5 mg/kg body weight showed carcinoma without mitosis with diffuse necrosis (Figure 6F, arrowheads).

**Figure 7:**
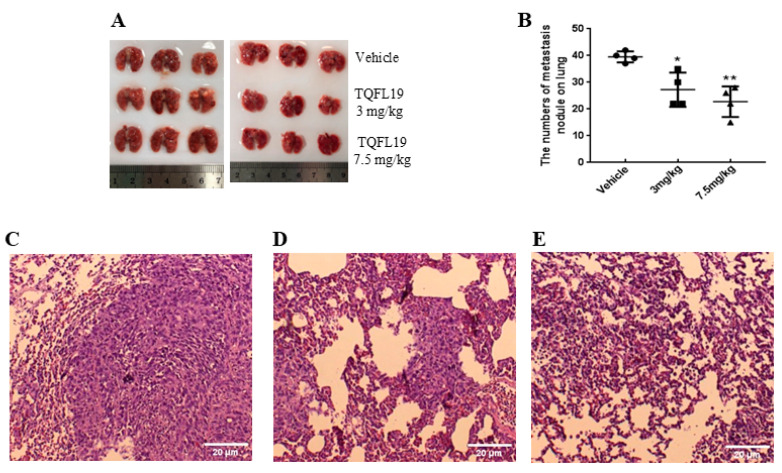
Metastasis of breast cancer cells to the lungs. (**A**) Morphology of lungs excised from control and mice treated with TQFL19 at concentrations of 3 and 7.5 mg/kg body weight. (**B**) Number of metastasis nodules in the lungs of control and mice treated with TQFL19 at concentrations of 3 and 7.5 mg/kg body weight. (**C**) Lung histopathology of control mouse group. (**D**) Lung histopathology of mouse group treated with 3 mg/kg body weight. (**E**) Lung histopathology of mouse group treated with 7.5 mg/kg body weight. * *p* < 0.05 and ** *p* < 0.01. Scale bar = 20 μm.

Metastasis of BC cells in the lungs was significantly diminished in mice that received treatment with 3 mg/kg (*p* < 0.05) and 7.5 mg/kg (*p* < 0.01), showing a dose-dependent response (Figure 7A,B). The images of tumors in the lungs treated with TQFL19 (Figure 7D,E) showed fewer tumor sizes than those of the vehicle-treated group (Figure 7C). Therefore, our in vivo results demonstrated that TQFL19 significantly inhibited BC cell migration.

## 3. Materials and Methods

### 3.1. Ethical Statement

Experiments using mice were carried out following the guidelines for animal care established by the institution and the protocols that received approval from the Southwest Medical University Ethical Committee, with approval code 20160086 (3 September 2016).

### 3.2. Reagents and Cell Lines

TQ was acquired from Sigma-Aldrich, (St. Louis, MO, USA). The CCK8 assay, fetal bovine serum (FBS), DMEM, and RPMI 1640 medium were sourced from Gibco Co. (Detroit, MI, USA). The specialized medium for MCF-10A cells was obtained from Saizhe Biological Technology Co. (Shanghai, China). The Annexin V/propidium iodide (PI) staining kit was purchased from BD Biosciences (San Jose, CA, USA). BALB/c mice were procured from Tengxin Biotechnology Co. (Beijing, China). Antibiotics (penicillin-streptomycin), trypsin-EDTA, and 4% paraformaldehyde were obtained from Beyotime Biological Technology Co., Ltd. (Shanghai, China). All TNBC cell lines, including BT549, MDA-MB-231, and 4T1, were sourced from ATCC (Manassas, VA, USA) and maintained at 37 °C with 5% CO_2_ in a medium containing 10% FBS.

### 3.3. Chemical Synthesis and Characterization and Purity Analyses

Synthesis of TQFL19 (molecular formula: C_17_H_15_C_l2_NO_2_) started with TQ by the following two steps. The amino group was introduced into the C-6 of TQ, and then the target compound was synthesized by a Schiff base reaction. First, the ethanol mixture of TQ (1 mM, 0.164 g) and sodium azide (1.3 mM, 0.084 g) was dissolved in ethanol and then 3 mL glacial acetic acid was added, and the reaction system was refluxed for 6 h. Volatiles were removed in vacuo and then purified via silica gel column chromatography. It was eluted with a mixture of petroleum ether and ethyl acetate in a 30:1 ratio to yield 3-amino-thymoquinone (NTQ) (0.116 mg). The reaction was repeated multiple times, and finally, 1.16 g NTQ was obtained. Then, an equal molar amount of NTQ (0.179 mg, 1 mM), 3,5-dichloro benzaldehyde (1 mM) 20 mL anhydrous ethanol, and 0.3 mL concentrated hydrochloric acid as a catalyst was added and stirred at 80 °C for 8 h. Then, the reaction solution was filtered, concentrated, and recrystallized with ethanol to obtain the target compound TQFL19 (0.241 mg).

Nuclear magnetic resonance (NMR) spectra for ^1^H and ^13^C were obtained using a Bruker AV-400 NMR spectrometer and AV-500 NMR (Bruker Co., Billerica, MA, USA), with tetramethyl silane serving as the internal standard. The chemical shifts (δ) and coupling constants (J) were reported in parts per million (ppm) and Hertz (Hz), respectively. NMR data were reported as follows: s = singlet, d = doublet, t = triplet, m = multiplet. High-resolution mass spectrum (HRMS) was obtained using electrospray ionization (ESI) and the quadrupole tandem time-of-flight (QTOF) mass analyzer (X500R, AB Sciex Co.; Framingham, MA, USA). The ESI parameters were as follows—temperature, 500 °C; ion source gas 1 and 2, 50 psi; and curtain gas, 30 psi. The MS spectra were collected by TOF-MS mode with the settings—ionspray voltage, 5500 V; CAD gas, 7 psi, declustering potential, 80 V; collison energy, 10 V, full scan mass range: 100–2000 Da, and accumulation time, 0.15 s.

### 3.4. Prediction of ADMET Properties

ADMET properties of TQ and TQFL19 were predicted by ADMET descriptor, Ames’s toxicity prediction, and molecular properties calculation present in BIOVIA Discovery Studio 2016 Client software. (https://www.3ds.com/products/biovia/discovery-studio, accessed on 13 December 2024). The pharmacokinetic properties of TQ and TQFL19 were analyzed using SwissADME server [20].

### 3.5. Cell Counting Kit-8 (CCK8) Assays

To determine cytotoxicity and half-maximal inhibitory concentrations (IC50s) of TQ and TQFL19, cells were plated in a 96-well plate at a density of 3000 to 5000 cells per well. They were exposed to varying concentrations of 0, 2.5, 5, 10, 20, 40, and 80 µM of TQ or TQFL19 for 24 and 48 h. After the treatments, 10 µL of CCK8 reagent was added to each well and incubated at 37 °C for 2 h. Following the incubation phase, the absorbance at 450 nm was assessed using a microplate reader. All experiments were repeated three times.

### 3.6. Apoptosis and Cell Cycle Assays

Assays for apoptosis and cell cycle analysis were performed using 4T1 or MDA-MB-231 cells with concentrations of 0, 2.5, and 5 μM of TQFL19. All experiments were performed three times to ensure consistent and reliable results.

### 3.7. Cell Growth, Migration, and Invasion Assays

The 16-well E-plates were utilized for cell growth, containing 100 µL of cells at a concentration of 1 × 10^4^ cells/mL in each well. CMI plates were used to assess cell migration and invasion indices. The lower chambers of the plates were filled with media containing 10% serum to stimulate chemotaxis. In contrast, the upper chambers were populated with additional cell suspensions at a density of 1 × 10^4^ cells/mL. Matrigel was applied in 1 × PBS to the CMI plates for the purpose of assessing cell invasion. Following 8 h of growth, the cells were treated with either 2.5 or 5 µM of TQFL19 or with dimethyl sulfoxide (DMSO) as a control. The growth, migration, and invasion of the cells were tracked and evaluated using a real-time cell analyzer [21]. All experiments were repeated three times.

### 3.8. Mouse Allograft Model

The 4T1 mouse cells were introduced into the mammary fat pads of female BALB/c mice to create a BC allograft model. Four days following the cell injection, the mice were randomly assigned to three groups, each consisting of six mice, and treated with either 0, 3, or 7.5 mg/kg of TQFL19. The tumor sizes were continuously monitored every 5 days. After a 30-day injection of 4T1 cells and a preceding 26-day TQFL19 treatment, the mice were sacrificed. The tumor tissues were then extracted and weighed.

Following the completion of the treatment, we dissected the mice’s lungs and counted the colony numbers formed within them. This process allowed us to assess and estimate the impact of TQ and TQFL19 on the migration and invasion of tumor cells.

### 3.9. Histopathology Assessment

Tumor tissues were preserved in 4% paraformaldehyde for 24 h, then embedded in paraffin and cut into sections 5 µm thick. Following this, the slides were dewaxed and hydrated using a series of xylene and alcohol solutions. The sections were stained with hematoxylin and eosin (H&E) and subsequently dehydrated again using the xylene and alcohol series. To complete the process, a coverslip was applied with one or two drops of neutral gum to ensure the slides were sealed and free of air.

### 3.10. Statistical Analysis

The statistical comparisons were performed using one-way ANOVA with Graph Pad Prism 6. A *p* value of less than 0.05 was regarded as significantly different.

## 4. Discussion

**Scheme 1:**
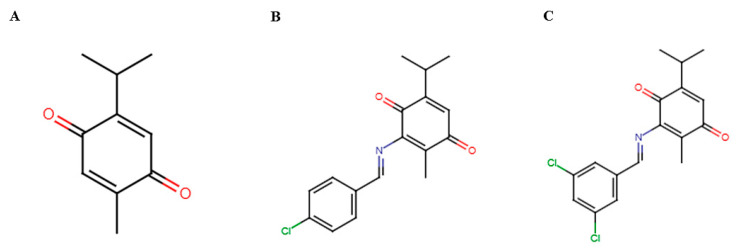
Structures of (**A**) thymoquinone (TQ), (**B**) TQFL12, and (**C**) TQFL19.

Natural bioactive compounds are crucial nutraceutical elements obtained from plants. These phytochemicals function as nutrients that help protect against diseases and provide a range of benefits, including antioxidant, anti-inflammatory, hypotensive, anti-aging, and anticancer properties [22]. TQ (Scheme 1A) has been thoroughly studied in relation to multiple types of cancer, such as pancreatic, breast, colon, liver, cervical, and leukemia [23,24]. Several studies investigated the cytotoxicity, apoptosis induction, and reduction in growth, migration, and invasion of BC cells [17].

In the current study, TQ induced cytotoxicity and apoptosis of 4T1, BT-549, and MDA-MB-231. This anticancer effect of TQ against TNBC was also recognized in our previous studies of Khan et al. [25] and Khan et al. [17]. TQ inhibited the TNBC cells’ growth, migration, invasion, and metastasis. Previous studies elucidated the antiproliferative, anti-migratory, and anti-metastatic effects of TQ through targeting of TWIST1, Interleukin 17 receptor D, cyclin-dependent kinase-2, heat shock protein family A member 6 zinc finger E-box binding homeobox-1, and others [19,21,25].

However, our newly synthesized derivative, TQFL19 (Scheme 1C), exhibited more significant anticancer effects compared with TQ through induction of more cytotoxicity on 4T1, BT-549, and MDA-MB-231 with less cytotoxicity to the normal breast cell (MCF10A). The same results were elucidated in our previous study on TQFL12 (Scheme 1B), another novel derivative of TQ [19]. Additionally, TQFL19 significantly reduced cell growth, migration, and invasion in murine 4T1 and human MDA-MB-231 cells, with a rapid response observed in 4T1. This effect might be due to the expression levels of antiproliferative, anti-migratory, and anti-metastatic proteins in each cell. Ünal et al. [26] stated that the parent molecule, TQ, suppressed pathways related to cell migration/invasion and angiogenesis in TNBC, including Integrin-*β*1, vascular endothelial growth factor, matrix metallopeptidase-2, and matrix metallopeptidase-9.

The process of cell division is managed by systems that guarantee the formation of two cells with identical genetic material. Checkpoints can pause the cycle or activate cell death when faced with permanent DNA damage [27]. Cyclin-dependent kinases (CDKs) work together with cyclins, p53, and cyclin-dependent kinase inhibitors (CKIs) to maintain a critical balance in processes such as cell growth, division, differentiation, and programmed cell death [28]. TQ-induced cell cycle arrest of MDA-MB-231 at G0/G1, [29], G2/M phase [30,31], and S phases [29,32]. In the current study, TQFL19 induced a 10% change in the G1-S phase of MDA-MB-231. An in vivo mice allograft model has been used to determine the effect of drugs on tumor size, tumor weight, and tumor histopathology [33]. TQ, the parent molecule of TQFL19, exhibited anti-metastatic and anti-migratory effects against TNBC mice models and significantly reduced tumor weight and tumor sizes [34,35]. The present study demonstrated that TQFL19 led to a significant reduction in tumor size, weight, and histopathological changes in a dose-dependent manner when compared with TQ. Also, TQFL19 significantly reduced the metastasis of 4T1 cells to the lungs in a dose-dependent manner. Similarly, TQFL12, a new synthetic derivative of TQ developed in our laboratory, was effective in suppressing tumor growth and metastasis in a mouse model using cancer cells while exhibiting reduced toxicity in comparison with TQ [19].

## 5. Conclusions

The data obtained from the current study demonstrates that TQFL19 exhibits promising anticancer properties by effectively impeding the growth, migration, and metastasis of BC cells in both in vitro and in vivo models. Furthermore, TQFL19 demonstrated the ability to hinder the spread of BC cells to lung tissues. More research is needed to explore the precise mechanisms behind TQFL19’s anticancer properties and its possible synergistic effects with chemotherapy drugs, as this is vital for comprehensively understanding its effectiveness in treating BC.

## Figures and Tables

**Table 1:** ADEMT, Ames’s toxicity, and molecular properties, and pharmacokinetics of Thymoquinone (TQ) and TQFL19.

		Thymoquinone (TQ)	TQFL19
**ADEMT**	**Solubility** (log mol/L)	−3.021	−5.902
	Solubility level	3	2
	AD-MET-BBB (log BB)	0.006	0.639
	PPB prediction	True	True
	Hepatotoxicity prediction	True	True
**Ames’s toxicity**	Ames’s prediction	Non-Mutagen	Non-Mutagen
	Ames’s probability	0.329	0.330
	Ames’s score	−11.192	−11.160
**Molecular properties**	H-donors	0	0
	H-acceptors	2	3
	Rotatable bonds	1	3
	Rings	1	2
	Aromatic rings	0	1
	Polar surface area	0.179	0.179
**Pharmacokinetics**	GI absorption	High	High
	P-gp substrate	No	No
	CYP1A2 inhibitor	No	Yes
	CYP2C19 inhibitor	No	Yes
	CYP2C9 inhibitor	No	Yes
	CYP2D6 inhibitor	No	No
	CYP3A4 inhibitor	No	Yes
	Log *K*p (skin permeation)	−5.74 cm/s	−5.29 cm/s

BBB, blood–brain barrier. PPB, human plasma protein binding.

**Table 2:** The IC_50_ values of TQFL19 and TQ in breast cancer cell lines.

	IC_50_ Values at 24 h (μM)		IC_50_ Values at 48 h (μM)	
Cell lines	TQFL19	TQ	TQFL19	TQ
4T1	26.169	72.05	15.478	32.916
BT549	44.271	>80	37.222	>80
MDA-MB-231	28.15	48.76	27.30	73.21
MDA-MB-468	57.23	>100	40.00	83.16

Each experiment was repeated three times.

## Data Availability

Data are contained within the article and Supplementary Materials.

## Supplementary Materials

The following supporting information can be downloaded at: https://www.mdpi.com/article/10.3390/molecules30040773/s1, Figure S1: ^1^H NMR spectrum of the novel compound TQFL19; Figure S2: ^13^C NMR spectrum of the novel compound TQFL19.

## Abbreviations

CDKs	Cyclin-dependent kinases.
CKIs	cyclin-dependent kinase inhibitors.
DMSO	dimethyl sulfoxide.
ESI	Electrospray ionization.
NMR	Nuclear magnetic resonance.
NTQ	3-amino-thymoquinone.
QTOF	quadrupole tandem time-of-flight.
TNBC	Triple-negative breast cancer.
TQ	Thymoquinone.
