# Supplementary figures and images for: TQFL19, a Novel Derivative of Thymoquinone (TQ), Plays an Essential Role by Inhibiting Cell Growth, Metastasis, and Invasion in Triple-Negative Breast Cancer
Source: Molecules. 2025 Feb 7;30(4):773. doi: 10.3390/molecules30040773 (PMC11858164; doi:10.3390/molecules30040773)

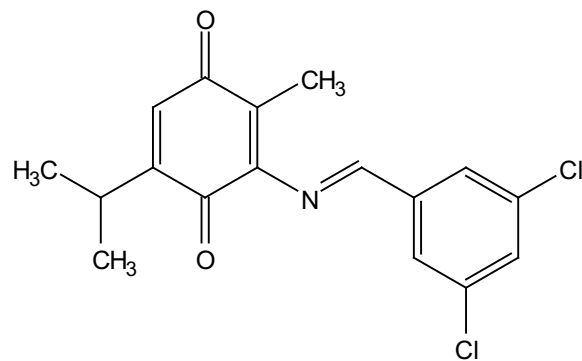

**TQFL19**

$^1\text{H}$  NMR, DMSO- $d_6$ , 400 MHz

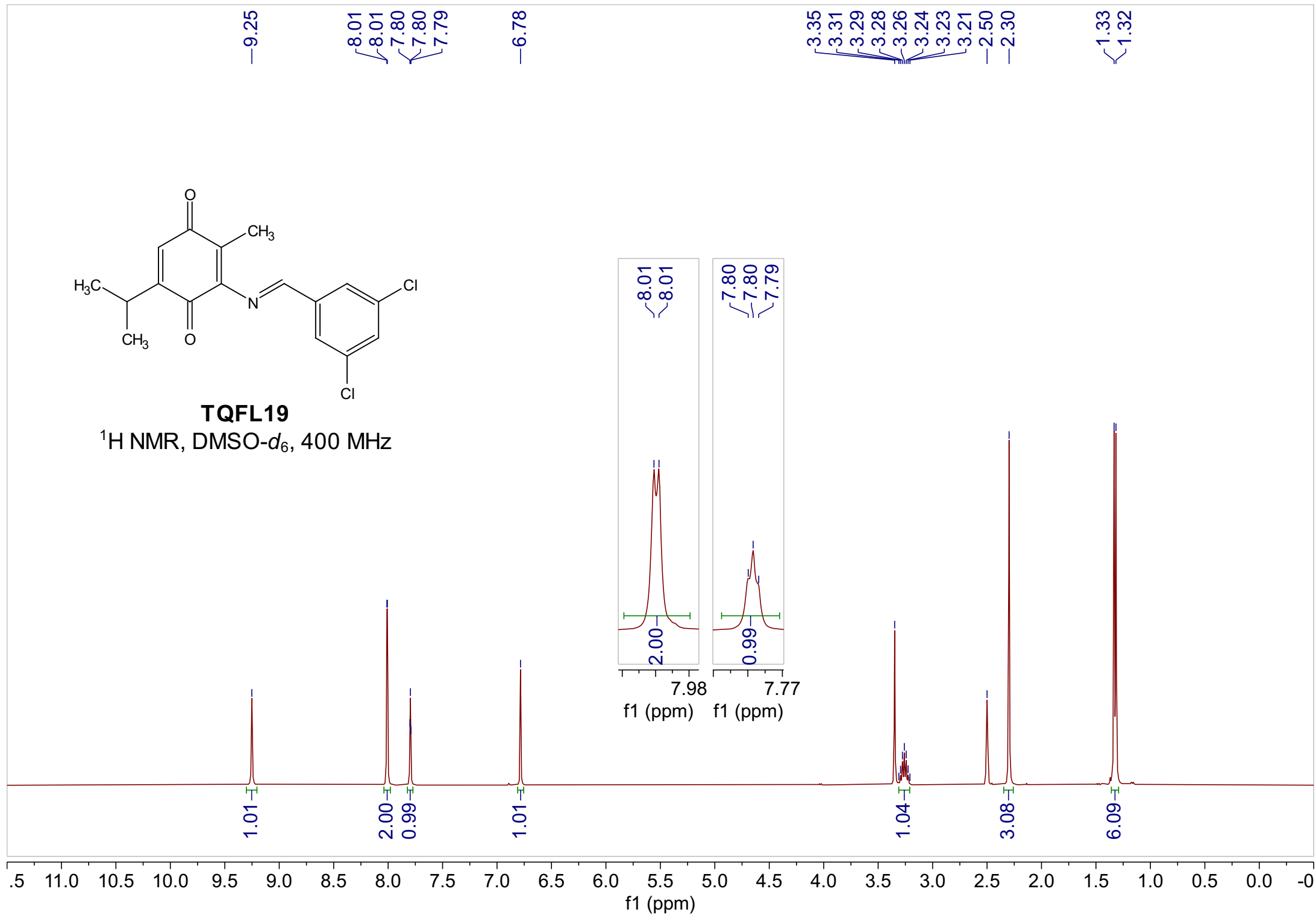

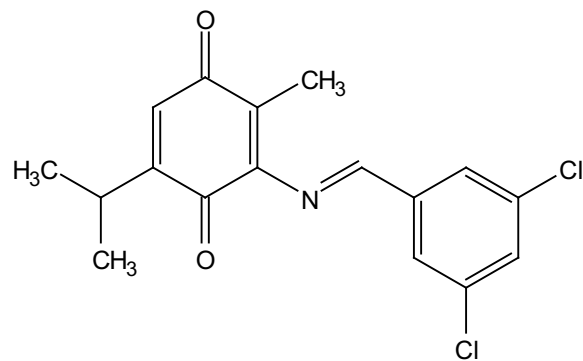

**TQFL19**

<sup>13</sup>C NMR, DMSO-*d*<sub>6</sub>, 101 MHz

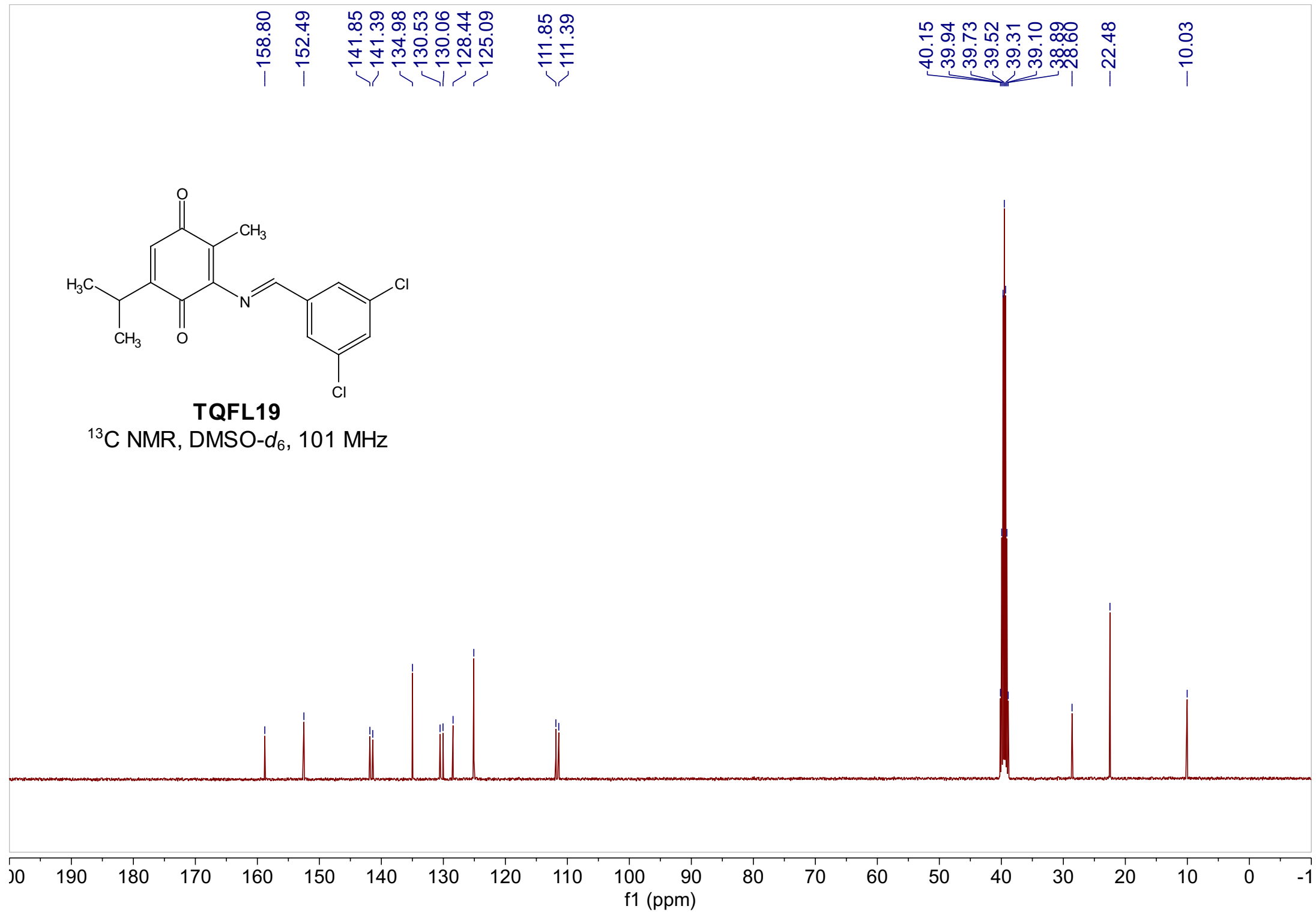

Supplement: Supplementary file 1 [file molecules-30-00773-s001.zip › molecules-3380136-supplementary.pdf]
